# Understanding malarial retinopathy: key predictors of cerebral parasite sequestration in an autopsy cohort

**DOI:** 10.1038/s41433-024-03329-1

**Published:** 2024-09-17

**Authors:** Owen K. Banda, Alice Muiruri Liomba, Kyle J. Wilson, Karl B. Seydel, Terrie E. Taylor, Nicholas A. V. Beare

**Affiliations:** 1 Malawi-Liverpool-Wellcome Trust Clinical Research Programme, Blantyre, Malawi; 2 Blantyre Malaria Project, Blantyre, Malawi; 3 Department of Eye & Vision Sciences, University of Liverpool, Liverpool, UK; 4 Department of Osteopathic Medical Specialities, Michigan State University, East Lansing, MI USA; 5 St. Paul’s Eye Unit, Royal Liverpool University Hospital, Liverpool, UK

**Keywords:** Parasitic infection, Eye manifestations, Prognostic markers

Malarial retinopathy (MR) is a unique set of fundus signs which have diagnostic and prognostic value in paediatric cerebral malaria [1]. Retinal examination is usually performed by ophthalmologists. However, there are just 2.5 ophthalmologists per million people in sub-Saharan Africa compared to a global mean of 31.7 [2]. Currently, there are insufficient ophthalmologists to support the scaling up of fundal assessments in children with cerebral malaria. Training of non-ophthalmologists to recognise the features of MR could bridge this gap.

The three major signs of MR are retinal haemorrhages, whitening and vessel discolouration. Haemorrhages had been previously described by researchers using direct ophthalmoscopy, but whitening and vessel change, which are specific to severe malaria, are better seen by indirect ophthalmoscopy through dilated pupils [3]. The ability of each of these signs to individually predict cerebral parasite sequestration remains to be established. This has implications for the training of non-ophthalmologists to recognise MR.

We hypothesised that identification of retinal haemorrhages alone may be insufficiently sensitive to predict cerebral parasite sequestration. This study aimed at identifying key predictors of cerebral parasite sequestration among the three major signs of MR in an autopsy cohort of Malawian children.

Paper and electronic records were reviewed for 103 autopsy cases, 88 with eye examination and 53 with sequestration on histologic examination. Data was collected on presence of cerebral parasite sequestration and presence or absence of each of the three major findings of MR (as determined by an ophthalmologist). The R package testCompareR [4] was used to calculate commonly used performance metrics and confidence intervals for each of the signs as a predictor of cerebral parasite sequestration. The results are shown in Table 1.

**Table 1:** Diagnostic metrics for the three major signs of malarial retinopathy.

Findings	Sensitivity	Specificity	PPV	NPV
Haemorrhages	66.7 (52.6–78.4)	82.1 (67.5–91.2)	82.1 (72.8–88.8)	66.7 (56.3–75.7)
Whitening	88.9 (76.7–95.4)	86.5 (72.3–94.3)	88.9 (80.4–94.1)	86.5 (77.6–92.4)
Vessel changes	69.2 (53.7–81.6)	100 (90.2–100)	100 (95.2–100)	73.9 (62.9–82.7)

*PPV* positive predictive value, *NPV* negative predictive value.

Among MR signs, haemorrhages are most easily identified even when using direct ophthalmoscopy [3]. However, our data suggests that haemorrhages alone are insufficient to accurately predict cerebral parasite sequestration. Conversely, though often absent (NPV = 73.9%), the presence of retinal vessel changes was a perfect predictor of cerebral sequestration (PPV = 100%) in the autopsy cohort. In the presence of peripheral parasitaemia, retinal vessel changes could be considered pathognomic of cerebral parasite sequestration. However, further work is required to validate this finding in a cohort that includes survivors. Retinal whitening has good NPV (88.9%) and PPV (86.5%).

Given that both whitening and vessel change are better predictors of cerebral parasite sequestration than haemorrhages alone, it is essential that fundoscopy training for non-ophthalmologists equips them to identify these signs. Vessel changes are often peripheral and are best visualised using indirect ophthalmoscopy. A low-cost indirect ophthalmoscope has recently been developed for use in resource-limited settings [5].

Our data suggests that, though readily identifiable, retinal haemorrhages cannot be substituted for comprehensive fundus examination in comatose children in malaria-endemic areas. The high reported sensitivity and specificity of MR appears to be driven by whitening and vessel change [3]. Training for non-ophthalmologists in identifying MR should be sufficiently comprehensive to ensure they can recognise these signs. Upskilling of the workforce may help to relieve some of the burden in areas where trained ophthalmologists are few. Additionally, effective upskilling of non-ophthalmologists to perform comprehensive fundoscopy could have implications for other common conditions such as diabetic retinopathy, where most physician-patient interactions are with non-ophthalmic specialists.

## Data Availability

The data that supports the findings of this study are available upon request from the corresponding author.

## References

[1] Beare NAV. Cerebral malaria—using the retina to study the brain. Eye. 2023;37:2379–84. 36788363 10.1038/s41433-023-02432-z PMC10397347

[2] Dean WH, Buchan JC, Gichuhi S, Faal H, Mpyet C, Resnikoff S, et al. Ophthalmology training in sub-Saharan Africa: a scoping review. Eye. 2021;35:1066–83. 33323984 10.1038/s41433-020-01335-7 PMC8115070

[3] Beare NAV, Taylor TE, Harding SP, Lewallen S, Molyneux ME. Malarial retinopathy: a newly established diagnostic sign in severe malaria. Am J Trop Med Hyg. 2006;75:790–7. 17123967 PMC2367432

[4] Wilson KJ, Roldán-Nofuentes JA, Henrion MYR. testCompareR: an R package to compare two binary diagnostic tests using paired data [version 1; peer review: awaiting peer review]. Wellcome Open Res. 2024;9:351. 10.12688/wellcomeopenres.22411.1 39502862 10.12688/wellcomeopenres.22411.4 PMC11535492

[5] Kousha O, Ganesananthan S, Shahin B, Ellis J, Blaikie A. Comparative evaluation of a new frugal binocular indirect ophthalmoscope. Eye. 2023;37:160–2. 34949786 10.1038/s41433-021-01901-7 PMC9829681

